# Mechanical interactions between bacteria and hydrogels

**DOI:** 10.1038/s41598-018-29269-x

**Published:** 2018-07-18

**Authors:** Nehir Kandemir, Waldemar Vollmer, Nicholas S. Jakubovics, Jinju Chen

**Affiliations:** 1 School of Engineering, Newcastle University, Newcastle Upon Tyne, NE17RU UK; 2 The Centre for Bacterial Cell Biology, Institute for Cell and Molecular Biosciences, Newcastle University, Newcastle upon Tyne, NE24AX UK; 3 School of Dental Sciences, Centre for Oral Health Research, Newcastle University, Newcastle upon Tyne, NE24BW UK

## Abstract

Mechanical interactions between bacterial cells and extracellular polymeric substance are essential in determining biofilm assembly and disassembly as well the mechanical characteristics of biofilms. However, the physics of these mechanical interactions in different cell culture conditions are poorly understood. We created typical artificial biofilm consisting of planktonic bacteria and hydrogel, in the absence of metabolic or regulatory effect. We have demonstrated that the cell culture medium can significantly affect the mechanical interactions between bacterial cells and hydrogels. The stiffness of the bacteria-hydrogel artificial biofilm cannot be simply attributed by the summation of the contribution from the bacteria and hydrogel based on the mathematical models and computational models. We have revealed that the tryptone component of Luria-Bertani broth medium plays an important role in stiffening effect of bacteria-hydrogel construct. Such significant stiffening effect can be explained by the following mechanism: the presence of tryptone in cell culture medium may enable the bacteria itself to crosslink the hydrogel polymer chains. Our findings have also demonstrated the synergy of modelling and innovative experiments which would potentially impact the biofilm control strategies.

## Introduction

All living things interact with their external environment and are susceptible to changes when the environment changes. Many studies have been conducted on animal cells to establish how they are affected by changes in the extracellular matrix (*i.e*. the environment), either in terms of two-dimensional interactions with surfaces^1–3^ or when cells are surrounded by an external 3D environment^4–9^. The impact of these environments on cell metabolism, protein synthesis, cytoskeletal architecture, cell motility, gene expression and cell mechanical properties have been studied^10–13^. Bacterial adhesion on surfaces has been widely investigated in many contexts including biomaterial associated infections, for example, since these interactions are considered to be the starting point for colonisation and biofilm formation^14–18^. Interactions of bacteria with many different surfaces have been studied including hard surfaces such as metals^18,19^, and different types of hydrogels^20,21^ that are natural, synthetic or a combination of both. Due to their aqueous environment, controllable mechanical and chemical properties and porous structure, hydrogels are suitable environments to interact with bacterial cells^22,23^. The chemical^24,25^ and physical properties^24,26–29^ of hydrogels, and environmental factors^24,30^ have been shown to affect bacterial growth rate and their ability to adhere. However, there is a lack of studies on how bacterial cells may be affected by a 3D micromechanical environment^31^ such as when bacteria penetrate soft tissues to form localised or systemic infections. There are several studies on embedding bacterial cells for 3D printing applications combining multiple types of bacterial cells to study cell-cell interactions^32^ and a controlled spatial distribution and concentration^33^ but the information on the mechanical interactions between the bacterial cells and hydrogels is limited. Therefore, this study aimed to investigate how physical (*i.e*. stiffness) and chemical (*i.e*. chemical composition of the growth environment) factors affect the mechanical interactions between bacterial cells and hydrogels, and bacteria cell mechanics when they are encapsulated in a 3D micro-environment. Due to the increased use of hydrogels in biomedical applications such as in implants (*i.e*. hydrogel coated cochlear implants)^34^ or indwelling medical devices (*i.e*. hydrogel coated venous catheters)^35^, understanding these properties might help us to design new materials or to select the most appropriate materials based on their interactions with bacterial cells. In addition, bacteria encapsulated in hydrogels have been used as artificial biofilm models^36–38^, which could simulate some important physicochemical characteristics of real biofilms and enable more reproducible results than the real biofilms. Therefore, it is essential to study the mechanical interactions between bacteria and hydrogels.

## Results and Discussion

### Stiffness of 1% agarose hydrogels without encapsulation at different strains

Agarose hydrogels were chosen as the matrix material due to their biocompatibility, inertness, gelling behaviour and the large content of water which favours bacterial cell hydration. In addition, previous work has demonstrated that agarose as a matrix polysaccharide was appropriate to simulate the extracellular polymeric matrix^39^. The stiffness of 1% agarose gels made with phosphate buffered saline (PBS), bacterial culture media (Luria-Bertani Broth (LB)) or distilled water has been reported in several studies^23,40–42^. These values were obtained from different mechanical tests, including simple tensile or compression tests, rheological characterisation or atomic force microscopy (AFM), and the range of the reported values varied between 14 kPa – 60 kPa. In addition to different constituents used and the various testing techniques, mechanical properties of agarose hydrogels also depended on the preparation protocols^42^.

**Figure 1:**
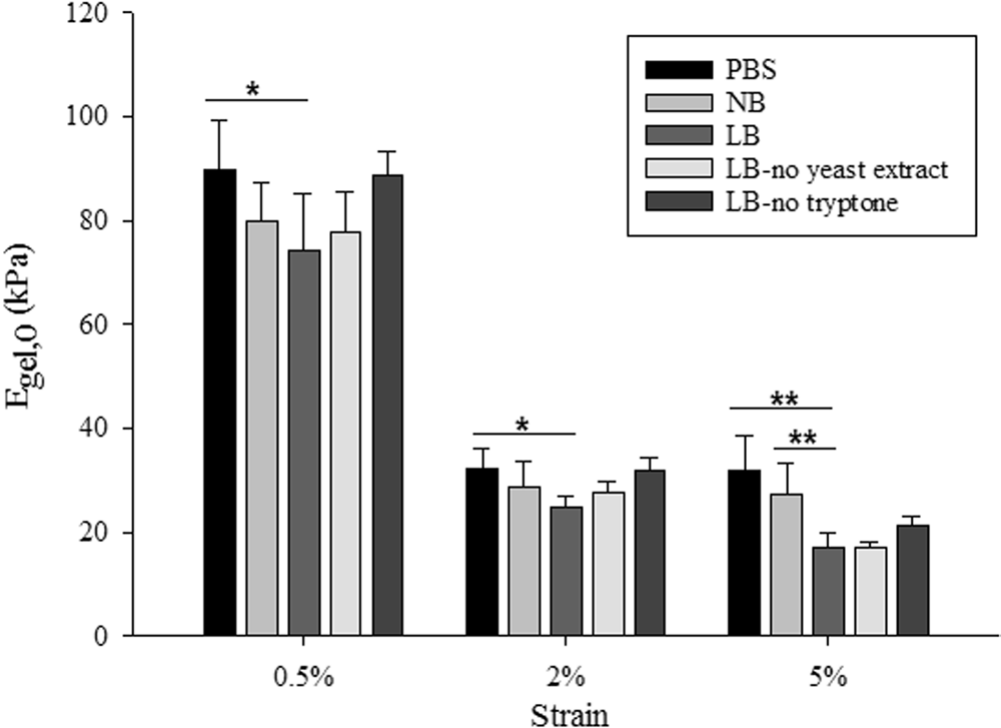
Instantaneous elastic moduli of 1% agarose hydrogels without encapsulation obtained from Hooke’s law. The symbols on the plot * and ** indicate $\,p\le 0.05$ and $p\le 0.01$ respectively between two different gels (at 0.5% and 2% strains only PBS – LB gels showed significant differences and at 5% strain both PBS – LB gels and NB – LB gels showed significant differences). Data represented as mean ± standard deviation, $n\ge 5$.

Measuring the stiffness of hydrogels infused with different growth media or buffer without bacterial encapsulation provided the basis of the measurements as the stiffness values measured were compared to the stiffness of gels with encapsulation (below). The instantaneous elastic moduli of 1% agarose hydrogels without encapsulation were obtained (Fig. 1) based on Hooke’s law (*i.e*. instantaneous modulus = maximum stress (*σ)* divided by the strain (*ε*)). When the loading time is much shorter compared to the relaxation characteristic time constant, Hooke’s law is a reasonable approximation at the given strains in this study. For all tested strains, 1% agarose gels made with PBS were stiffer than the gels made with growth media, suggesting that the medium used to form the hydrogel affects the stiffness of the gel. When the stiffness of the gels made with buffer and culture media (*i.e*. LB and NB) were compared, PBS gels were stiffest, followed by gels made with NB and then those made with LB. For all applied strains, PBS gels were significantly stiffer than LB gels (*p* < 0.05 for 0.5% and 2% applied strain and *p* < 0.01 for 5% applied strain) but there were no significant differences observed between PBS and NB gels. At lower strains (0.5% and 2%), there were no statistically significant differences between the stiffness of gels made with LB or NB. By contrast, at 5% strain, gels containing NB were significantly stiffer than those containing LB (*p* < 0.01).

A strain dependent behaviour of stiffness was observed for the tested hydrogels. For instance, the apparent stiffness of LB gels decreased with the increasing applied strain, *i.e*. the stiffness of the gel was obtained as 74.2 kPa, 24.8 kPa and 17.1 kPa when the applied strain was increased from 0.5% to 2% and 5% respectively. As reported previously^43–45^, several polymers have shown strain softening effect similar to the classical model of rubber elasticity, where the apparent stiffness of the material decreases with the increasing applied strain.

For PBS, NB and LB gels without encapsulation, there was a significant decrease (*p* < 0.01) in stiffness values when the applied strain was changed from 0.5% to 2%, which could be due to the collapse of the porous structure of the hydrogels, similar to what has been reported in many other porous materials^46–48^. However there were no significant differences (*p* > 0.05) in the measured stiffness when strain changes from 2% and 5%, which suggests that the stress-strain curve of these hydrogels reached a plateau in this strain region.

It was evident that the presence of tryptone weakened the hydrogel as measured at different applied strains (see Fig. 1). Interestingly, the stiffness of hydrogels made with LB, LB-no tryptone or LB-no yeast extract decreased by 76–78% when the applied strain increased from 0.5 to 5%. For PBS and NB gels, the stiffness decreased only by 66% when the applied strain increased from 0.5 to 5%.

### Stiffness of hydrogels with bacteria

**Figure 2:**
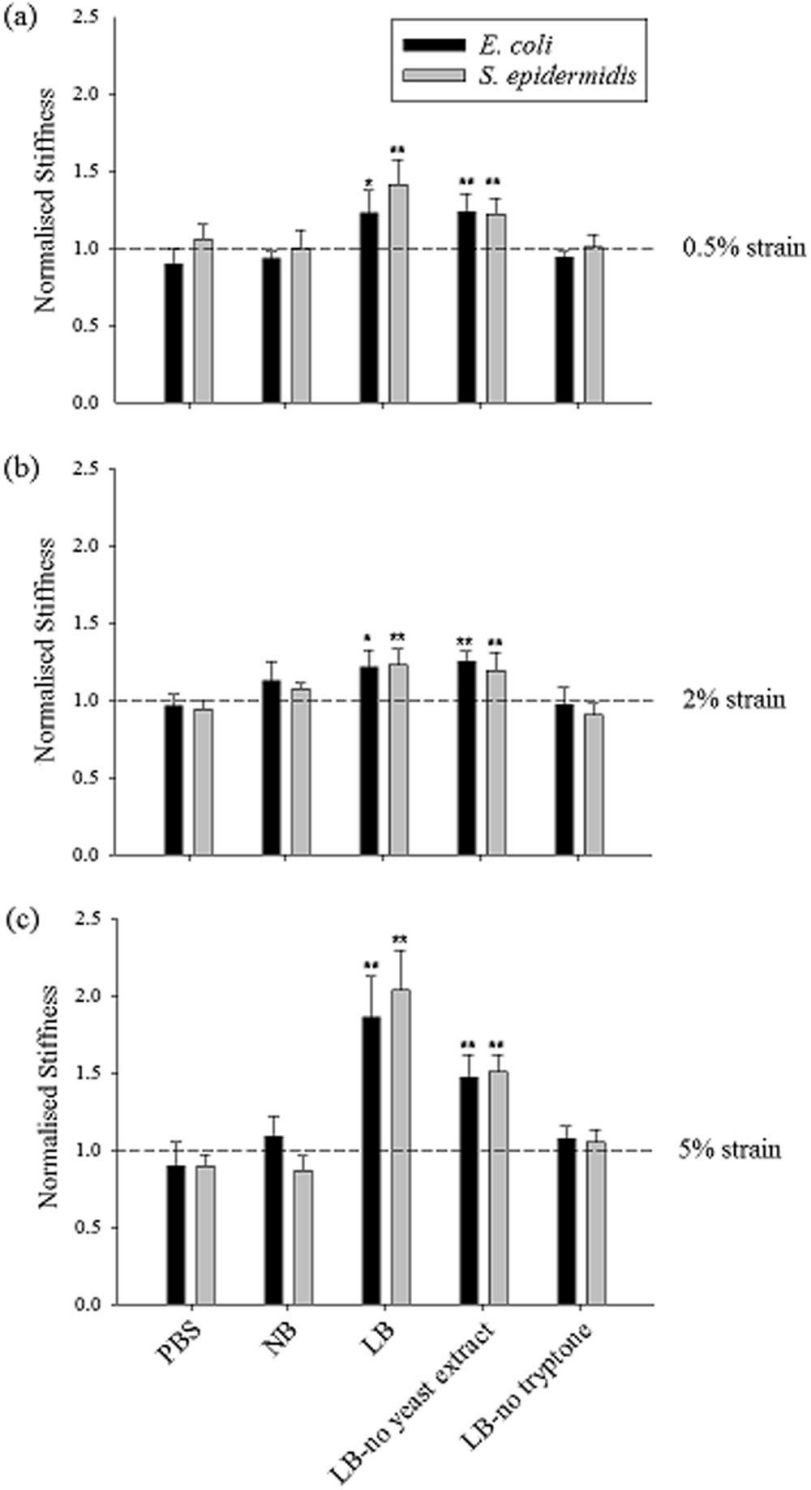
Normalised stiffness values of different LB-based hydrogels when (**a**) 0.5% strain, (**b**) 2% strain and (**c**) 5% strain were applied (the bars represent the gels with bacteria, the dashed line shows the normalised value for gels without encapsulation and error bars represent standard deviation). This normalisation represents the fold change in the stiffness in hydrogels containing encapsulated cells compared with those without cells. The symbols on plots * and ** indicate $\,p\le 0.05$ and $p\le 0.01$ respectively between the gels made with the same liquid media/buffer with and without encapsulation of bacterial cells, determined from 3 independent experiments.

Having measured the stiffness of hydrogels alone, the next step was to assess whether the encapsulation of bacteria in the gels led to any measurable changes in the stiffness. Therefore, *E. coli* or *S. epidermidis* were encapsulated at a concentration equivalent to 1% of the total hydrogel volume. A similar stiffness characterisation was carried out for gels with encapsulated cells and the obtained stiffness values were normalised by the corresponding gel without encapsulation. The stiffness values of gels with encapsulated bacterial cells are given in Table S1. 1% LB gels with encapsulated *E. coli* and *S. epidermidis* cells were stiffer than 1% LB gels without bacteria. Interestingly, for the 1% gels made with PBS and NB, such an increase in stiffness was not observed and they showed similar stiffness values as the gels without bacteria (Fig. 2a–c). The significantly higher stiffness of LB gels with encapsulated bacteria compared with LB gels without bacteria could be attributed to the interactions between the bacterial cells and the media used to prepare the hydrogel. To investigate this further, experiments were performed to determine which constituent of LB medium, tryptone or yeast extract, was causing this increase in stiffness. Different media were prepared in which either constituent was omitted, for LB-no yeast extract gels yeast extract was omitted and for LB-no tryptone gels tryptone was omitted. The salt content was not changed since NaCl was present also in NB and PBS, and therefore could not be solely responsible for the observed differences in hydrogel stiffness between agarose formulations. The stiffness of these gels with and without bacteria was calculated and normalised. Only LB-no yeast extract gels with bacteria showed significant increases in normalised stiffness when bacterial cells were encapsulated, which possibly suggests that bacterial surface properties may have been altered in response to the peptides present in tryptone (Fig. 2a–c). Both types of bacterial cells (*E. coli* – rod shaped and *S. epidermidis* – spherical shaped) behaved in a similar pattern suggesting that different bacteria interact similarly with the media and the applied mechanical stimuli.

Previous studies have shown that, bacterial cells can adhere to soft surfaces more easily than to harder surfaces^27,28^. This is in agreement with our observations showing that bacterial cells interact differently with the 1% LB and LB-no yeast extract hydrogels, which were softer than the other hydrogels tested before encapsulation. It has also been found that mechanical properties of agarose gels are affected by the presence and amount of sugars (such as glucose and sucrose), urea and guanidine hydrochloride^49,50^. Although the peptide-based tryptone does not have well-defined chemical composition, the different peptides within tryptone could affect the mechanical properties of an agarose gel in a similar manner.

The force generated by bacterial cells might cause a change in pore size in agarose gel, resulting in differences in the overall stiffness of the bacteria/hydrogel composite. However, such a mechanism in altering pore size of agarose gel is unlikely to be wholly responsible for the dramatic stiffening effect of some bacteria/hydrogel composites as seen in Fig. 2, provided the fact that the volume fraction of bacterial cells is so low (up to 1%). A significant increase in hydrogel stiffness is usually caused by chemically induced cross-linking of polymer chains that form the hydrogel network^51^. Particle reinforced hydrogels have also been reported elsewhere. For example, it was observed that bioactive glass particles can increase the stiffness of the polysaccharide gellan gum hydrogel by ~ 100 times at 2% concentration of particles^52^. Such a huge stiffening effect cannot be explained by the composite theory but could indicate crosslinking of the loose polymer chains by the particles. In this study, a similar mechanism may apply where bacterial cells crosslink the loose polymer chains. Also, it has also been demonstrated that the stiffness of material can affect the biological activities of bacterial cells when these cells are seeded on the material surface^27,53^ and such an effect can take place when bacterial cells are encapsulated inside hydrogels as well.

### Computational modelling in comparison to mathematical models

Composite materials are made by combining two or more materials having different properties in a way that they do not dissolve or blend in each other to obtain a new material with unique properties^54^. In this sense, hydrogels with encapsulated cells or particles may be treated as composites providing the mentioned composite characteristics. In the literature, there are several mathematical models, including the Voigt model, Reuss model, and Hashin and Strikman model with upper and lower bounds (*i.e*. limits), to determine composite stiffness^54–56^. These models were employed to establish whether the experimental data and the simulation data would match at the tested volume of 1%.

A computational model was developed to represent the compression tests for the case where the encapsulated particles do not interact with their environment (*i.e*. inert particles). Several variables were considered in this model, namely the applied compressive strain, the volume fraction of particles, Young’s modulus and Poisson’s ratio of the particle and the matrix, so that the model correctly represented the compression test conditions. It was reported in a previous study^57^ that the whole and live *E. coli* cells have a Young’s modulus of 2-3 MPa. This stiffness value was approximately 200 times as the hydrogel Young’s modulus^41^. The individual matrix and particle stiffness values were chosen so that they represented the same fold difference between the particle (bacteria) and the matrix (hydrogel). The same fold difference was also employed for the mathematical models. Providing a similar applied strain value and volume fraction of particles (1%) allowed comparisons with the experimental results. Young’s modulus was calculated based on the force value applied to the composite when the applied strain value reached 5%.

An incompressible material has a Poisson’s ratio of 0.5^58^. When hydrogels are fully swollen their properties resemble rubber like materials^59^ which are highly incompressible and have a Poisson’s ratio close to 0.5. Similarly, bacterial cells are rigid structures featuring viscoelastic properties^23^ and their adapted Poisson’s ratio were documented between 0.4–0.5 in several studies^60–63^. Therefore, in the Hashin and Strikman models and in the computational model, both the matrix and the particle Poisson’s ratios were selected as 0.45.

**Figure 3:**
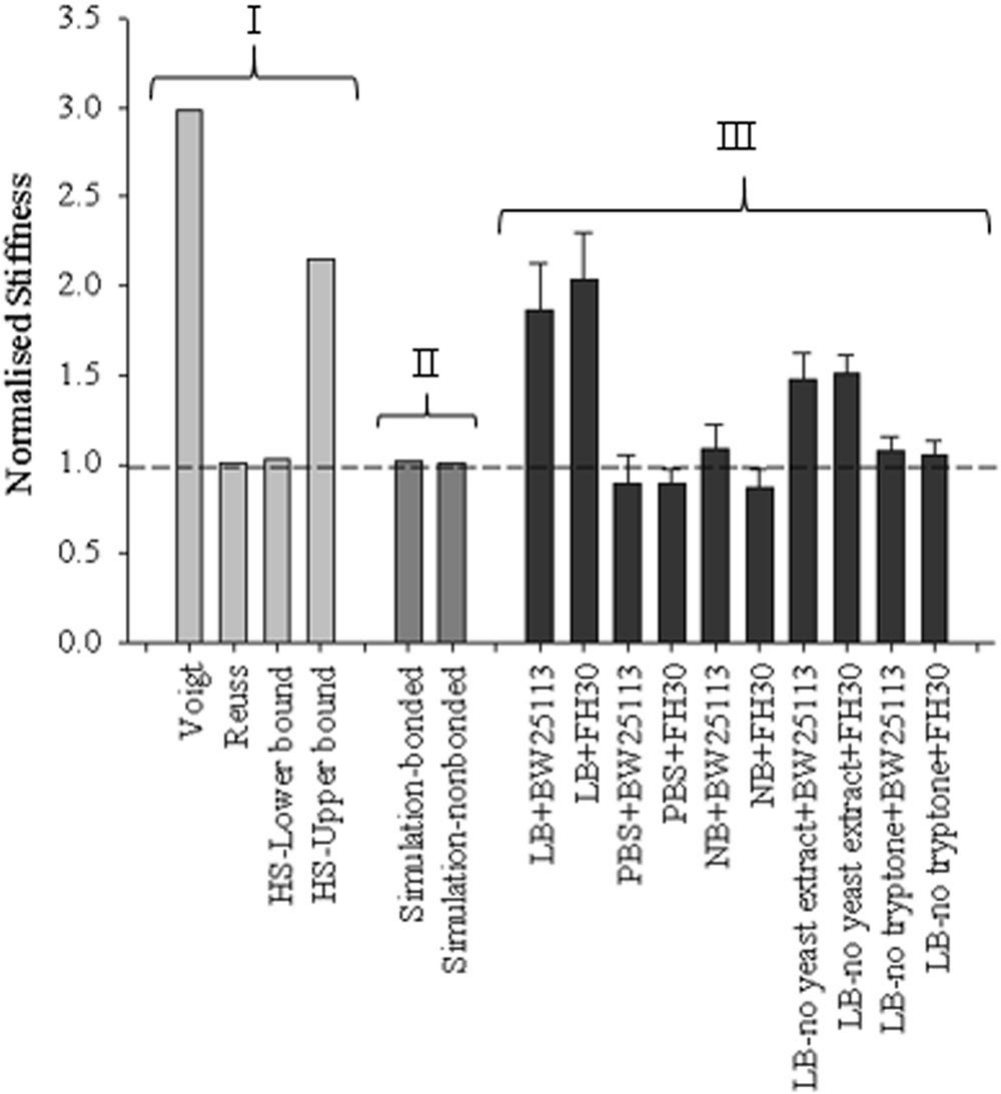
Normalised stiffness values obtained from mathematical models (group I - Voigt model, Reuss model, and Hashin and Strikman model with upper and lower bounds), simulations (group II) and experiments (group III) respectively for 5% applied strain. All the data were normalised by either the adapted gel stiffness, *i.e*. matrix without particles and separation (for simulations), or the corresponding 1% agarose gel stiffness with the same media/buffer (for experimental data). The dashed line represents the case without particles/encapsulated cells and the volume fraction of particles is 1%.

The mathematical model values, simulation values and the experimental data obtained from compression tests were plotted together for comparison (Fig. 3). The results obtained from both bonded and non-bonded simulation were almost completely coincident with the Reuss model/HS-lower bound. There were two different groups of experimental data at 1% volume fraction: the first group was located between Voigt model/HS-upper bound and Reuss model which consisted of data points from LB and LB-no yeast extract gels (where significant differences were observed between the gels with and without encapsulation) and the second group was closer to Reuss model/HS-lower bound which consisted of data points belonging to 1% gels made with PBS, NB and LB-no tryptone (where no significant differences were observed between the gels with and without encapsulation).

The data obtained from the simulations represented the case for inert particles, where they were not affected by different media or possible structure irregularities of the gel. However, the experimental data suggested that the interactions between the bacterial cells and their environment caused the data to agree with different mathematical models which represent different alignment of the particles. All the simulation results and most of the experimental data agreed with Reuss model and HS lower bound model, except the experimental data for bacteria encapsulated hydrogels made with LB or LB-no yeast extract.

### Bacteria growth in 1% agarose gels made with different growth media

**Figure 4:**
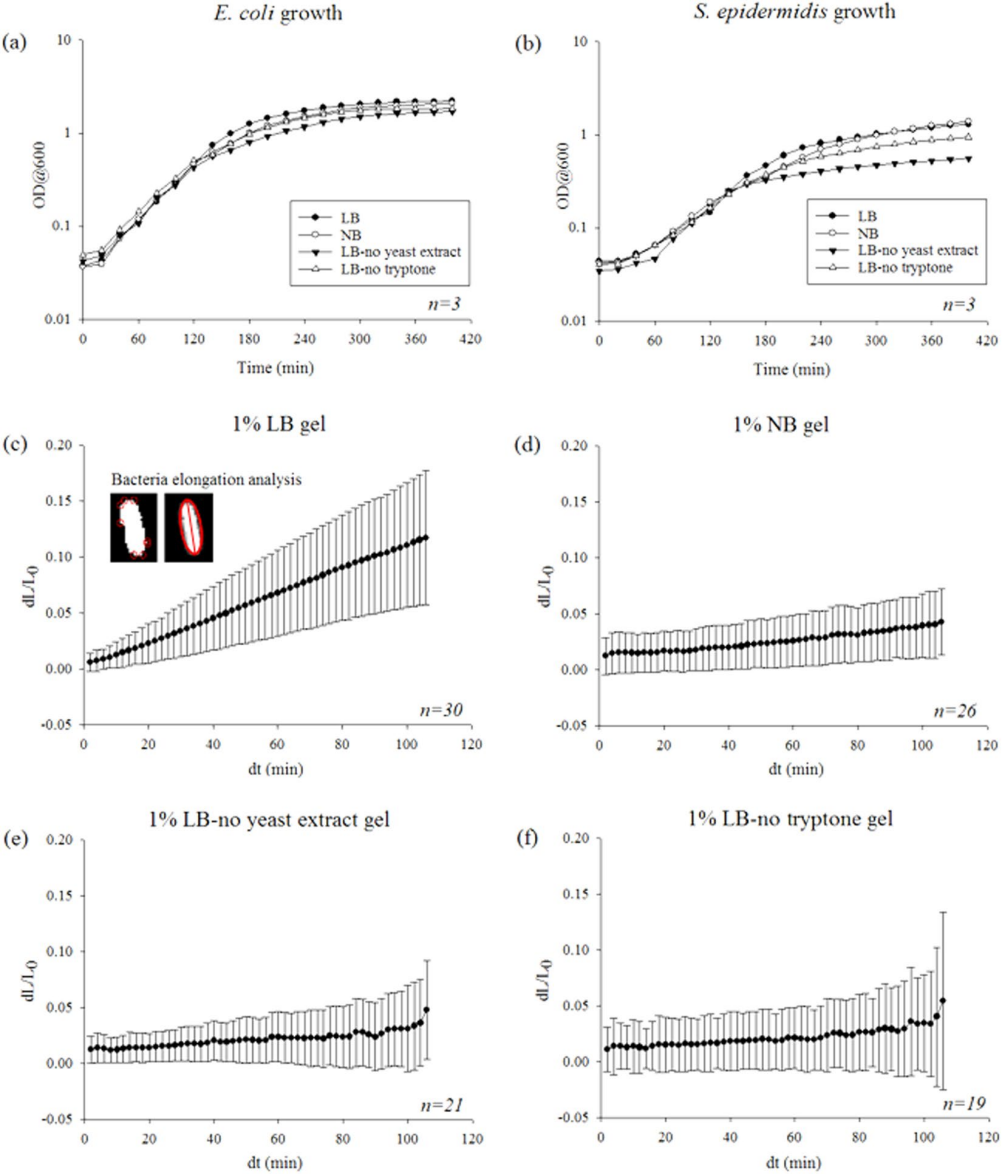
(**a**) *E. coli* and (**b**) *S. epidermidis* cell growth in different liquid growth media. Stationary phase *E. coli* cell elongation in 1% agarose hydrogels made with (**c**) LB, (**d**) NB, (**e**) LB-no yeast extract and (**f**) LB-no tryptone. Data points and error bars represent mean values and standard deviation, respectively. Number of replicates for each experiment is indicated at their individual sections. The images embedded in (**c**) illustrates how the bacteria elongation was determined: an ellipse was fitted around the identified edges of the bacteria and the major axis of the ellipse indicated the length of the bacteria. The same approach was used for all of the images captured at different times and the differences between the axis lengths indicated the bacteria growth at a particular time interval.

Batch growth curves were obtained for *E. coli* and *S. epidermidis* in the growth media used to make the hydrogels (LB, NB, LB-no yeast extract and LB-no tryptone, respectively) to obtain their growth behaviour in an environment where no hydrogel forces were acting on them (Fig. 4a,b). During exponential phase there were no significant differences between growth rates when different growth media were used for both *E. coli* and *S. epidermidis* cells. However, the lag phase of *S. epidermidis* cells was longer compared to that of *E. coli* cells for all the growth media considered. Bacteria growth in liquid media did not affect the overall growth behaviour of the bacterial cells when the growth took place without any forces acting on them by the hydrogels (*i.e*. when they were not encapsulated). The doubling time for *E. coli* cells was measured as 20 min and the doubling time for *S. epidermidis* cells was measured as 40 min during exponential phase.

To determine how bacterial cells interacted with the hydrogels, to demonstrate their viability when encapsulated and to examine the effect of different media, bacterial growth in hydrogels was measured (Fig. 4c–f). First of all, it is important to point out that bacterial cell lysis was not observed. The change in length of the bacterial cells were normalised by their initial length to account for variability of the initial bacterial cell size. The length of the bacterial cells at each time point was represented by the length of the major axis of an ellipse fitted around the edges of the bacteria (see the images of individual cells embedded in Fig. 4c). After the imaging period of two hours, *E. coli* cells had elongated approximately 12.8% ± 1.1% in LB gels, 4.3% ± 0.58% in NB gels, 4.8% ± 0.96% in LB-no yeast extract gels and 5.5% ± 1.8% in LB-no tryptone gels (data given as mean ± standard error). There were no significant differences in cell elongation rate for NB, LB-no yeast extract and LB-no tryptone gels. However, the rate of bacteria elongation in LB gels was significantly higher than the rate obtained from other hydrogels (*p* < 0.05). This may be due to LB medium being nutritionally richer compared to the other growth media. It also suggests that either yeast extract or tryptone alone cannot play a dominant role in cell growth. Both of them need to be present in the medium to promote cell growth.

In addition, the doubling time of the encapsulated cells were calculated by fitting a curve to the data. From the fit, doubling time of *E. coli* cells in LB gels, NB gels, LB-no yeast extract gels and LB-no tryptone gels were calculated as 15.2 h, 60.5 h, 80.3 h and 69.4 h respectively. The reason for the long doubling time may be due to the bacterial cells not being able to reach exponential phase or grow at all due to the reaction forces acting on them from their 3D micro-environment, especially when certain growth media were used. It may also be limited by nutrient diffusion, which presumably will be much slower in a hydrogel than in broth. As the bacterial cells have such slow growth rates in hydrogels made with different media, it is unlikely that volume changes caused by growth in media could have led to significant increases in the stiffness of the bacteria/gel composites with the timeframe of the mechanical tests. Our computational simulations also confirmed this since the modelled encapsulated particles did not change their volume in time and the difference between the gel with particles and without particles did not show any significant differences for both bonded and non-bonded cases (see Fig. 3). Therefore, the key mechanisms to answer for this stiffening effect is likely due to cell-materials interactions when cells are in contact with the hydrogels with different physical/chemical properties. It is known that bacterial cells change surface characteristics to adapt to nutrient availability^64^ which would affect how they interact with their environments. In addition, different sources of tryptone can affect the physiological state of bacterial cells even in liquid medium^65^. Similar effects can apply to bacterial cells encapsulated inside hydrogels. In addition, it has been shown that different cell-materials interactions affected the cell membrane, cell phenotype and other physiological conditions of bacteria when bacterial cells are in contact with two-dimensional materials surfaces^66^. All these may also happen when bacterial cells are in contact with three-dimensional materials.

## Conclusions

In summary, this study employed an array of computational and experimental approaches to explore how the three-dimensional biomaterials can affect bacteria. It is found that the presence of tryptone tends to weaken agarose hydrogels. However, the presence of tryptone in culture medium apparently enhances the stiffness of the hydrogel when bacterial cells are present, possibly by enabling bacteria to crosslink the polymer chains in agarose. This may imply that tryptone is capable to trigger some unknown effect on bacterial surface characteristics. However, tryptone alone could not support cell growth unless yeast extract was also present in LB medium. The majority of previous work has only reported that the nutrient and materials stiffness would affect bacterial growth on materials surfaces. Our study revealed that biophysical parameters of materials can also regulate biophysical responses of bacteria that are encapsulated inside hydrogels. Such mechanisms may also be extended to biofilms because the hydrogel matrix mimics the extracellular polymer produced in a biofilm. Thus, changes in extracellular polymer matrix caused by factors such as chemicals or mechanical stresses may regulate bacteria mechanics, which potentially may lead to changes in cell structure and metabolic functions. Future studies will aim to investigate the impact of different 3D microenvironments on transcription and protein expression in bacteria, to better understand the potential for the control of biofilms by modulation of the extracellular matrix.

## Materials and Methods

### Bacterial stains and growth media

*E. coli* was routinely cultured in Luria-Bertani (LB) broth medium, consisting of tryptone (1% w/v), yeast extract (0.5% w/v) and NaCl (1% w/v) and *S. epidermidis* was routinely cultured in nutrient broth (NB) medium prepared from a commercially available mixture (Oxoid CM0067 Nutrient Broth No.2)^67^. PBS buffer (Lonza, 17–517Q), consisted of KH_2_PO_4_ (0.1440% w/v), NaCl (9% w/v) and Na_2_HPO_4_ (0.7950% w/v). Both growth media and the phosphate buffered saline (PBS) buffer were used to prepare agarose hydrogels. When required, modified version of LB growth medium were prepared by omitting one reagent, either yeast extract for ‘LB-no yeast extract’ or tryptone for ‘LB-no tryptone’. The pH of these media was adjusted to 7.5 ± 0.2 and they were autoclaved at 121 °C and a pressure of 1.2 bar for 15 min.

*E. coli* BW25113^68^ cells and *E. coli* MC1061^69^ pEGFP (plasmid kindly provided by Jörg Götz, University of Tübingen, Germany) were used as representatives of gram negative bacteria. *S. epidermidis* FH30 was isolated from a case of chronic rhinosinusitis^70^ and used as the representative of gram positive bacteria. Bacteria stock solutions were kept at −80 °C and the working cultures were obtained from single colonies on fresh agar plates prepared with the suitable growth media. For the *E. coli* BW25113 and *S. epidermidis* FH30 overnight cultures, 20 ml of growth media were inoculated with a single colony from a fresh plate and shaken at 37 °C, 160 rpm. For the selection of *E. coli* MC1061 pEGFP containing GFP plasmid, 100 µg/mL ampicillin was added to the growth media. After 16 h, cells were in stationary phase (OD600 ≈ 1.7–1.8). The procedure for preparing the overnight cultures to be used for the encapsulation process was the same for all types of hydrogels, providing similar bacterial cell activity when encapsulated.

### Preparation of hydrogels and encapsulation of bacterial cells

Hydrogels were produced from low gelling 2-hydroxyethyl agarose type VII-A (Sigma-Aldrich). This type of agarose powder was selected due to its lower gelation temperature which enabled a longer working time at a liquid state. The gelation point of this type of agarose is specified as 26 °C ± 2 °C.

1% (w/v) agarose was prepared and autoclaved at 121 °C, 1.2 bar and cooled to ~35 °C. Both bacteria strains were cultured in their suitable growth media (*E. coli* cells in LB liquid media and *S. epidermidis* cells in NB liquid media) and then encapsulated them in different hydrogels. Therefore, prior to encapsulation, the activities of the bacteria were the same.

A cell pellet was obtained in a centrifuge tube with volumetric markings by centrifuging the overnight culture of both bacterial cells at 4,000 rpm for 10 min at 10 °C. Based on the volume of the pellet, hydrogel solution was added to achieve a 1% volumetric ratio of bacteria to hydrogel. Therefore, the bacterial cells occupied up to 1% of the total volume of the hydrogel prepared. The sterile agarose gel or the encapsulation solution was injected into a 12 × 10 × 3 mm mould, which was designed and manufactured to hold 8 individual cylindrical samples of 20 mm diameter and 3 mm thickness, and placed tightly on a glass slide having the same dimensions. After the injected hydrogel solution solidified completely, the mould and the glass slide were separated and the samples were collected inside a sterile container filled with the corresponding liquid medium or buffer to avoid drying. The samples were kept in the liquid at 4 °C for 3 h prior to mechanical testing for them to reach a fully hydrated state. The diameter of the fully hydrated hydrogel samples were 20 mm and the thickness of the hydrogels were 3 mm.

### Compression tests with stress relaxation

Compression tests with stress relaxation were carried out at room temperature using the EZ test machine (EZ-SX, Shimadzu, Japan) to determine the mechanical properties of hydrogels with and without encapsulation. In the tests, 0.5%, 2% and 5% strain values were used. These values were chosen since they were small strain values (*i.e*. the test samples were not harmed macroscopically after the compression tests) and there was a 10-fold difference between the lowest and the highest strain value which enabled a broad response range. A 0.05 N of pre-load was applied to the samples to determine the surface and the strain rate for all tested strains was 0.01 mm/s. In order to prevent dehydration of hydrogels during the tests, the gels were soaked in the medium or buffer they were made of and a specifically designed hood was used to shelter the gel. When compressed to the specified strain, the top plate of the test machine were held on the hydrogel sample for 30 mins and the force to maintain the strain were recorded over the test time where the samples showed stress relaxation behaviour. Usually, relaxation behaviour of viscoelastic materials can be captured by various viscoelastic models^11,71^. Different viscoelastic models (e.g. Prony series model and Burgers model) have been adopted for curve fitting, however, they failed to accurately capture the relaxation curves in this study. They only predicted the equilibrium modulus well. Although there are other more complicated models available^72^, it makes the data interpretation challenging. When the loading time is much shorter compared to the relaxation characteristic time constant of the hydrogel, the Hooke’s law was a reasonable approximation at the given strains in this study. Actually, it was found that the bacteria has little contribution to the overall viscous behaviour of the bacteria/hydrogel composite during the stress relaxation in this study. In such case, the instantaneous elastic modulus of either hydrogels or bacteria/hydrogel composite can be obtained either from the loading curve or the relaxation curve which should give the same results. When the Hooke’s law (*i.e. σ* = *Eε*) was adopted, the instantaneous elastic modulus of gel, *E*_*gel*.0_ was determined by using the true stress and strain values at the peak force (*F*_*gel*_) during the compression test. Similar principle applies to the bacteria/hydrogel composites. Therefore, the stiffness of the composite normalised to its hydrogel counterpart is given by,

1 $${E}_{normalised}=\frac{{E}_{composite,0}}{{E}_{gel,0}}=\frac{{F}_{composite}}{{F}_{gel}}$$

For different materials tested, at least 5 trials were carried out for each measurement. For the measurements with bacterial cells, different sets of samples were prepared and tested as 3 independent experiments.

### Computational modelling of compression responses

**Figure 5:**
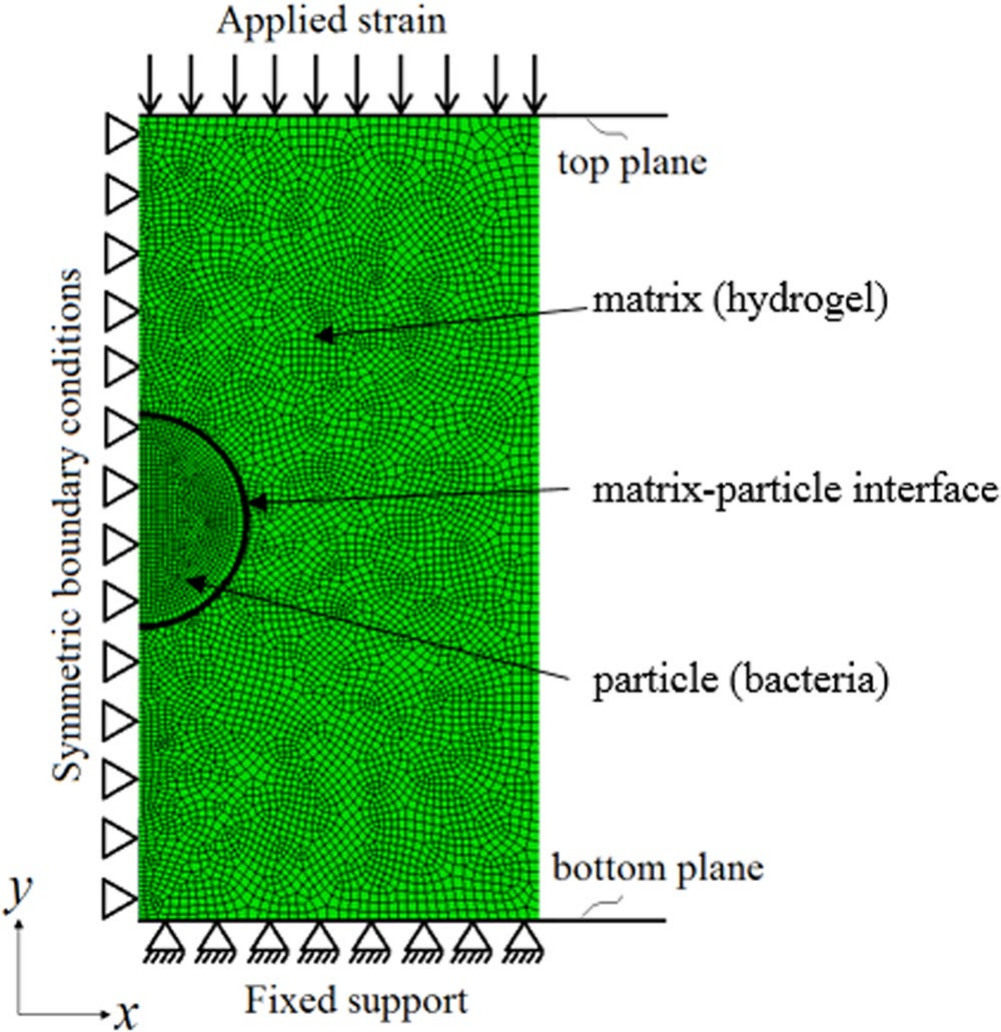
Representation of the model for the compression tests. Applied boundary conditions: symmetric boundary conditions on the matrix-particle structure and the planes provided us the opportunity to only model half of structure while restraining the linear movement in *x* direction and the rotational movement in *y* and *z* directions, fixed support applied at the bottom plane for the restriction of movement and a 5% strain was applied through the top plane. Mesh distribution of the matrix-particle structure: applied mesh is finer to correctly represent the matrix-particle interface and at the symmetry plane with an element size of 0.05, elsewhere the element size is 0.1. The element type used is CAX8R (An 8-node biquadratic axisymmetric quadrilateral with reduced integration).

A simple representative model was created using the commercial finite element software ABAQUS CAE 6.14 (Dassault Systèmes Smulia Corp. 2014, Providence, RI, USA) to carry out simulations to determine the Young’s modulus of the bacteria-hydrogel composites. The model used was a representation of the encapsulation with symmetric boundary conditions and a uniform static displacement of 5% was applied. The boundary conditions were applied in a way that they mimicked the compression tests where the bottom plane was fixed, the movement in the horizontal direction was limited and a vertical compression was applied at the top surface (Fig. 5). Two different boundary conditions at the interface between cell and hydrogel were considered: bonded and non-bonded. For bonded condition, the matrix (hydrogel) and the particle (bacteria) were deforming together when a specific strain was applied, *i.e*. the separation between the matrix and the particle was not allowed. For non-bonded case, the separation between the matrix and the particle was allowed. Different mesh sizes were applied on the model and the *E*_*composite*_ values were obtained until the obtained *E*_*composite*_ values were independent of the mesh size used. A finer mesh size was used at the matrix-particle interface and where the boundary conditions were applied. The mesh structure of the model is presented at Fig. 5. The results obtained from simulations and experiments were compared to several mathematical models used to determine composite stiffness: the Voigt model, Reuss model, and Hashin and Strikman (HS) model with upper and lower bounds^54–56^. The equations for the mathematical models are summarised in Table 1. The individual matrix and particle stiffness values were chosen so that they had a 200-fold difference between the particle and the matrix. For the mathematical models and the computational model, the particle volume fraction was chosen as 1% (similar to the volume fraction in experiments). The Poisson’s ratio for the particle and matrix was 0.45. HS shear moduli and bulk moduli equations shown in Table 1 refer to the HS upper bounds. To obtain HS lower bounds, the indices (1 and 2) were reversed.

**Table 1:** Mathematical model equations. In the equations, subscript 1 refers to the properties of matrix and subscript 2 refers to the properties of particle.

**Model**	**Equation**	
***Voigt***	${E}_{Voigt}={E}_{1}{V}_{1}+{E}_{2}{V}_{2}$	${V}_{1}+{V}_{2}=1$
***Reuss***	${E}_{Reuss}=\frac{{E}_{1}{E}_{2}}{{E}_{1}{V}_{2}+{E}_{2}{V}_{1}}$	
***HS – shear moduli***	${G}_{HS}={G}_{2}+\frac{{V}_{1}}{\frac{1}{{G}_{1}-{G}_{2}}+6({B}_{2}+2{G}_{2}){V}_{2}/[5(3{B}_{2}+4{G}_{2}){G}_{2}]}$	$G=\frac{E}{2(1+\upsilon )}$ $B=\frac{E}{3(1-2\upsilon )}$
***HS – bulk moduli***	${B}_{HS}={B}_{2}+\frac{{V}_{1}({B}_{1}-{B}_{2})(3{B}_{2}+4{G}_{2})}{(3{B}_{2}+4{G}_{2})+3({B}_{1}-{B}_{2}){V}_{2}}$	

*E*, *G, B, V* and ν refer to Young’s modulus, shear modulus, bulk modulus, volume fraction and Poisson’s ratio, respectively.

### *E. coli* cell elongation analysis

Stationary phase fluorescent *E. coli* MC1061 pEGFP cells were encapsulated in the 1% agarose hydrogel made with the growth medium and the resulting hydrogel was injected in an isolated PDMS chamber to avoid drying of the gel. Using a confocal microscope equipped with PFS (Nikon A1R), cell elongation was imaged for two hours at 37 °C with 60× oil immersion lens. A 20 µm range was selected for imaging so that it was located at the middle of the chamber and away from chamber boundaries, and contained enough cells to be analysed. After obtaining the time lapse images, they were analysed to determine the extent of cell elongation in gels with different growth media using an in-house MATLAB code. We carefully avoided choosing the bacterial cells which were clustered with other cells or adjacent to another cell. The edges of the chosen single bacterium were determined, an ellipse was fitted around the identified edges, and the length of the major axis which specified the length of the bacteria was determined. This procedure was applied for all the images taken at 2 min intervals for 2 h for the same bacterial cell.

### Statistical analysis

Analysis of Variance (ANOVA) with Tukey’s post hoc test was applied to determine the statistical differences between hydrogels with and without encapsulation and of gels made with different growth media or buffer. The significance level (*α*) was taken as 0.05.

### Data availability

The datasets generated and analysed during the current study are available from the corresponding author on reasonable request.

## Electronic supplementary material

Table S1
