# Supplementary material for: Mechanical interactions between bacteria and hydrogels
Source: Sci Rep. 2018 Jul 18;8:10893. doi: 10.1038/s41598-018-29269-x (PMC6052062; doi:10.1038/s41598-018-29269-x)
Supplement: Supplementary file 1 — Table S1 [file 41598_2018_29269_MOESM1_ESM.pdf]

Supporting Information

Mechanical interactions between bacteria and hydrogels

Nehir Kandemir[1], Waldemar Vollmer[2], Nicholas S. Jakubovics[3], Jinju Chen[1]*

[1]School of Engineering, Newcastle University, Newcastle Upon Tyne, NE17RU, UK; [2]The Centre for Bacterial Cell Biology, Institute for Cell and Molecular Biosciences, Newcastle University, Newcastle upon Tyne, NE24AX, UK; [3]School of Dental Sciences, Centre for Oral Health Research, Newcastle University, Newcastle upon Tyne, NE24BW, UK.

Corresponding Author

*Email: jinju.chen@ncl.ac.uk

Table S1. Instantaneous Young's moduli of 1% agarose hydrogels with encapsulation obtained from Hooke's law (Data represented as mean ± standard deviation, $n \geq 5$ for each independent experiment ($n_{ie} = 3$))

Applied strain (%)	Buffer or media	$E_{gel.0}$ (kPa) (*E. coli* encapsulation)	$E_{gel.0}$ (kPa) (*S. epidermidis* encapsulation)
0.5	PBS	81.0 ± 8.5	95.3 ± 8.4
	NB	74.6 ± 3.6	79.3 ± 10.2
	LB	91.2 ± 10.8	105.1 ± 11.6
	LB-no yeast extract	95.8 ± 9.4	95.0 ± 7.9
	LB-no tryptone	83.5 ± 3.6	89.7 ± 6.7
2	PBS	31.2 ± 2.4	30.4 ± 2.1
	NB	32.4 ± 3.6	30.8 ± 1.1
	LB	30.3 ± 2.7	30.6 ± 2.5
	LB-no yeast extract	34.6 ± 1.6	32.9 ± 3.2
	LB-no tryptone	30.8 ± 3.5	28.7 ± 2.2
5	PBS	28.6 ± 5.0	28.6 ± 2.5
	NB	29.6 ± 3.6	23.7 ± 2.6
	LB	31.7 ± 4.6	34.8 ± 4.3
	LB-no yeast extract	24.9 ± 2.5	25.5 ± 1.8
	LB-no tryptone	23.0 ± 1.7	22.5 ± 1.7
